# Evaluation of Serum Concentration of the Myokine Irisin (FNDC5) in Patients with Age-Related

**DOI:** 10.14744/bej.2021.52533

**Published:** 2021-09-27

**Authors:** Burak Turgut, Kadir Mercan, Nevin Ilhan, Selahattin Semih Aydogan

**Affiliations:** 1. Department of Ophthalmology, Onsekiz Mart University Faculty of Medicine, Canakkale, Turkey; 2. Department of Ophthalmology, Private Sevgi Hospital, Malatya, Turkey; 3. Department of Biochemistry, Firat University Faculty of Medicine, Elazig, Turkey; 4. Department of Ophthalmology, Yuksek Ihtisas University Faculty of Medicine, Ankara, Turkey

**Keywords:** Age-related macular degeneration, dry, FNDC5, irisin, serum level, wet

## Abstract

**Objectives:**

This study evaluated the serum irisin level of patients with age-related macular degeneration (ARMD) and compared it with that of healthy individuals.

**Methods:**

The serum irisin level of 15 healthy controls (Group 1) and 15 dry ARMD patients (Group 2) and 15 wet ARMD patients (Group 3) were measured using the enzyme-linked immunosorbent assay (ELISA) method and compared.

**Results:**

There was no statistically significant difference between the groups in terms of age or gender (p>0.05). The mean serum irisin levels of Group 1, Group 2, and Group 3 were 25.81±24.04 ng/mL, 22.93±19.05 ng/mL, and 12.38±8.16 ng/mL, respectively. Although the mean irisin level in the wet ARMD patients was lower than that of the control and dry ARMD groups, there was no statistically significant difference between the groups (p>0.05).

**Conclusion:**

The results suggest that the serum irisin level in ARMD patients is not different from that of healthy individuals. Studies of larger groups that examine the irisin level in the vitreous and neovascular membranes will further elucidate any role in the pathogenesis of ARMD.

## Introduction

Age-related macular degeneration (ARMD) is a chronic and progressive disease of the macula that causes vision loss in people over 50 years old in developed countries. In ARMD, neurodegeneration which affects photoreceptor cells, retina pigment epithelium (RPE), Bruch membrane, and choriocapillaris in the macular region develops (1, 2). ARMD is divided into two clinical types as dry (non-neovascular and non-exudative) and wet (neovascular and exudative) ARMD. Dry ARMD (d-ARMD) includes macular RPE changes and drusen in early stages and macular atrophy in late stages. In wet ARMD (w-ARMD), the choroidal neovascular membrane (CNVM) and RPE detachment are developed. The w-ARMD, which is seen in 10–15% of patients with ARMD, is responsible for about 90% of visual loss due to the complications associated with neovascularization (NV) (1, 2). Vascular endothelial growth factor-A (VEGF-A) is the main factor responsible for NV and increased vascular permeability in w-ARMD (3-6). At present, intravitreal administration of various anti-VEGF agents is the most important treatment in ARMD treatment, and it has been widely demonstrated that vision loss due to w-ARMD could be markedly prevented by this intervention (7).

Irisin is an exercise-induced, glycosylated protein that is defined as myokine and adipokine. It is formed by the proteolytic cleavage of fibronectin type III domain-containing protein 5 (FNDC5) in muscle tissue. Irisin turns white adipose tissue cells into brown adipose tissue cells through exercise. Elevated irisin level causes an increase in energy metabolism, weight loss, and heat in the body and improves glucose tolerance (8-10). Irisin was first discovered from mouse skeletal muscle, and it was shown that FNDC5 mRNA, the precursor of the irisin, was existed in various tissues or organs. In addition, irisin is released highly in the optic nerve and lower concentration in the retina (11). It has been reported that irisin immunoreactivity is present in the porcupine neuroretina (12). In another study, it has been demonstrated that irisin immunoreactivity was found in all layers of the retina excluding the outer nuclear layer in hamsters (13).

Recent reports demonstrated that serum or plasma levels of irisin are decreased in many patients with metabolic syndromes and related diseases (14). High plasma irisin levels are associated with the stage of diabetic retinopathy (DR) (15). It has been also suggested that irisin might protect against DR with potential anti-IL-17A effects (16). In addition, it has been reported that the patients with proliferative DR had decreased serum and vitreous irisin levels compared with the control group and type 2 diabetic patients without DR and that irisin levels were associated with the presence of diabetic nephropathy and DR (17).

To PubMed search, we could not detect a report on the serum irisin levels in any ophthalmic disease in the literature. In light of the recent data, we hypothesized that irisin might play a role in ARMD pathogenesis, and our study, we aimed to evaluate the levels of irisin in the serum in patients with ARMD.

## Methods

This pilot study was conducted in full accordance with the Declaration of Helsinki and approved by the ethics committee of the university (Date: 30.03.2017, Number: 16). All the study subjects were provided informed consent.

A full ocular examination containing best-corrected visual acuity, slit-lamp biomicroscopic examination, ocular tonometry, indirect ophthalmoscopy, fundus fluorescein angiography (FFA, Zeiss FF 450 IR plus), and optic coherence tomography (OCT, Zeiss Cirrus HD-5000) were performed for all study subjects. ARMD classification as d-ARMD or w-ARMD was performed by a single retina specialist.

The study included three groups: Group 1 included 15 sex- and age-matched healthy control subjects without drusen, RPE changes, or CNVM. Group 2 included 15 dry ARMD patients having macular RPE changes and/or drusen or geographic atrophy without CNVM detected by FFA and OCT. Group 3 included 15 wet ARMD patients with CNVM or disciform scar detected by FFA and OCT.

The patients with morbid obesity, and/or chronic alcohol abuse, and any hematological, psychiatric, immune, malignant, obvious inflammatory, systemic infectious, connective tissue, cardiac, renal, hepatic, cerebrovascular disease, cardiovascular disease (such as diabetes mellitus, systemic hypertension, etc.), and the patients with ocular infection and inflammation, retinal vaso-occlusive disease, and patients having retinal laser photocoagulation or intravitreal injection were not admitted to the study.

### The Assay of Serum Irisin Concentrations (18)

Blood samples were taken from patients and healthy controls to measure irisin levels at 09.00 h after overnight fasting in all subjects. All subjects were rested for 15 min before the blood collection process. Samples were delivered to the laboratory within 20 min. They were centrifuged in 2000 turns for 10 min at 4°C. The sera are stored at −80°C until biochemical assay. For serum irisin measurements, a commercial kit (Sunred Bio, Baoshan, Shanghai) was used. Serum irisin levels were assayed by enzyme-linked immunosorbent test (ELISA) following the manufacturer’s instructions. The minimum detectable level (sensitivity) was less than 0.157 ng/mL and the assay range for irisin was 0.2–60 ng/mL. Intra- and interassay CVs were less than 10% and 12%, respectively. All samples were measured spectrophotometrically using ELx800TM Absorbance Microplate Reader (BioTek Instruments, Inc., Winooski, VT, USA) at 450 nm. The biochemist blindly assayed samples. The results are presented as ng/mL.

### Statistical Analysis

Results are given as means±SD. The data provided from the subjects were entered into the Statistical Package for the Social Sciences, version 11.0 (SPSS Inc., Chicago, IL) for statistical analysis. Individual group parameters were assessed with the one-sample Kolmogorov–Smirnov Z test and were found to be abnormally distributed (p< 0.05). Hence, statistical comparisons between groups were performed by the non-parametric Kruskal–Wallis and the Mann–Whitney U-test. For all comparisons, statistical significance was defined by p<0.05.

## Results

There was no statistically significant difference between the groups in terms of age and gender (p>0.05). The mean serum irisin levels of Group 1, Group 2, and Group 3 were found 25.81±24.04 ng/mL, 22.93±19.05 ng/mL, and 12.38±8.16 ng/mL, respectively. Although the mean irisin levels in w-ARMD patients seem to be numerically lower than those in the control and d-ARMD groups, there was no statistically significant difference between the groups (p>0.05) (Table 1).

**Table 1.** Comparison of serum irisin levels between the groups

**Study groups**	**Subject Number**	**Serum Irisin**	**Standard deviation**	**p-value**	**Significance**
		**(ng/mL)**			**(Bonferroni corrected Mann–Whitney U test)**
Control	15	25.81	24.04	>0.05	d-ARMD versus w-ARMD
d-ARMD	15	22.93	19.05		Control versus d-ARMD
w-ARMD	15	12.38	8.16		Control versus w-ARMD

d-ARMD: Dry age-related macular degeneration; w-ARMD: Wet age-related macular degeneration.

## Discussion

Although there is some evidence that irisin promotes angiogenesis through stimulation endothelium cells migration and tubule formation (19) and these limited data are controversial, it has been speculated that possible protective mechanisms of irisin on the retina are local delivery of FNDC5, then irisin production, and transport into the central nervous system and brain-derived neurotrophic factor (BDNF)-producing retina cells of irisin, subsequently, the induction of BDNF expression through a Pgc1alpha pathway and last TrkB activation (20-22).

Recent studies have reported that irisin secreted the following exercise might be a beneficial role in brain function in neurodegenerative diseases such as Alzheimer’s disease (23-27).

Irisin is a peptide secreted by muscle and regulates energy metabolism. It has been well-known that BDNF plays an important role in synaptic functions and neuronal survival. It has been reported that exercise-induced irisin increases the efficacy of BDNF in the hippocampus. On the other hand, irisin prevents the ischemia-induced neuronal injury due to oxidative stress through a reduction in the secretion of a pro-inflammatory cytokine such as tumor necrosis factor-α. In addition, a recent study suggested that irisin has a neuroprotective effect through the suppression of ROS-NLRP3 inflammatory signaling, expression of interleukin-1β, and the activation of caspase 1 signaling in ischemic conditions (28-39).

Recently, Guler et al. (40) reported that irisin levels increased in the aqueous humor of patients with pseudoexfoliative glaucoma. In addition, we recently reported that serum irisin levels are not different in various open-angle glaucoma types (17). As it has been considered that low levels of chronic inflammation in choroid and complement system pathologies were played role in ARMD pathogenesis, irisin may be a potential candidate for ARMD treatment through the suppression of inflammatory processing (41-46).

In the setting of our study, we hypothesized that the levels of irisin in the serum in ARMD patients may be lower than those of healthy controls because of the possible tissue-protective effects of irisin on the retina and choroid. We found that there was no statistically significant difference between the groups concerning the mean serum irisin levels. Although its levels in w-ARMD patients seem to be numerically lower than those in the control and d-ARMD groups.

To the best of our knowledge, it seems like the first study investigating serum irisin level in ARMD patients. Although the main limitation of our study is the lower cohort number, our results suggest that serum irisin levels do not change in patients with ARMD. The insignificant results concerning the levels of serum irisin in our study may be due to a local neurodegeneration nature of ARMD. However, yet, in the light of literature, we speculate that irisin may be a protective myokine against ARMD by possible effects including suppression of the inflammation or neuroprotection on neural and vascular tissues. Thus, as a next step, the measurement of both vitreous and aqueous humor levels of irisin in ARMD patients with and without treatment may obtain detailed and realistic comments about the effects of irisin in ARMD patients. Further researches are needed to have more information on the effects of irisin and investigate the levels of free and bound irisin and determine the exact role of irisin in the pathogenesis of ARMD. Studies in which large groups are formed and iris levels in vitreous and neovascular membranes are measured are necessary to reveal whether iris plays a role in ARMD pathogenesis.

## Funding

We thank Bayer TÜRK KIMYA for supporting with providing the Irisin assay kits for this study.

## Acknowledgments

This study was conducted by BT, KM, NI and SSA. Biochemical assays were performed by NI. Collection of data, typing, and editing of the manuscript were performed by BT, KM and SSA.'' şeklinde olmalıdır. All authors reviewed and approved the final manuscript. The authors declare that they have no financial conflicts of proprietary interest with this work.

## Disclosures

### Ethics Committee Approval:

Firat University Faculty of Medicine Ethics Committee, protocol number: 16, Date: 30/03/2017.

### Peer-review:

Externally peer-reviewed.

## Conflict of Interest:

None declared.

## Authorship Contributions:

Involved in design and conduct of the study (BT, KM, NI, SSA); preparation and review of the study (BT, KM, SSA); data collection (BT, KM, SSA); and statistical analysis (BT, SSA).
